# Capitate Fracture with Scapholunate Ligament Rupture: A Case Report

**DOI:** 10.5704/MOJ.2603.023

**Published:** 2026-03

**Authors:** LR Cruz, RJ Ngo, JH Pua

**Affiliations:** Department of Orthopaedics, Jose R Reyes Memorial Medical Center, Manila, Philippines

**Keywords:** capitate fracture, open reduction internal fixation (ORIF), wrist injury

## Abstract

Capitate fractures are rare, accounting for 1–2% of all carpal fractures, and are frequently associated with high-energy trauma. When accompanied by scapholunate ligament rupture, the injury becomes significantly more complex with increased risk of instability, non-union, chronic instability. We report the case of a 36-year-old male police officer who sustained a high-energy wrist injury following a motorcycle accident. Imaging revealed a displaced transverse capitate fracture (AO 73A) with an associated scapholunate ligament rupture. The patient underwent open reduction and internal fixation (ORIF) of the capitate using a headless screw with ligament repair and dorsal capsular imbrication. Early postoperative rehabilitation was initiated with a structured and progressive program. At 10 weeks post-surgery, the patient demonstrated near-normal wrist function, range of motion, and grip strength. Radiographic follow-up confirmed fracture union and maintained carpal alignment. The patient successfully resumed full occupation duties without functional limitations. This case highlights the importance of timely diagnosis and comprehensive surgical management in complex carpal injuries involving both bony and ligamentous structures. Early mobilisation and a structured rehabilitation program played a pivotal role in achieving excellent functional outcomes, emphasising the critical integration of surgical and post-operative care in managing such injuries.

## Introduction

Fractures of the capitate comprise 1 – 2% of all carpal fractures and are due to high-energy trauma such as direct blow/crush, scaphocapitate syndrome, or anvil mechanism^1,2^. Given the capitate’s central location and its articulation with surrounding structures, injuries involving the capitate often occur alongside other bony or ligamentous injuries such as transscaphoid, transcapitate, or greater arc perilunate injury. On the other hand, isolated capitate fractures are typically nondisplaced and are managed conservatively with immobilisation^3^.

Most existing literature on capitate fractures are limited to complications due to neglect or inadequate management, thus posing a challenge^4^. At present, there is no standardised treatment algorithm for such complex injury. This report aims to contribute to the limited literature by detailing the clinical presentation, diagnostic work-up, surgical management, post-operative outcomes, and rehabilitation approach of such injury.

## Case Report

A 36-year-old right-handed male police officer presented with left-hand pain following a motor vehicle accident (MVA) after reportedly falling asleep while intoxicated. He initially sought consultation at a private orthopaedic clinic and was referred to our institution approximately 12 hours post-injury.

**Fig. 1:**
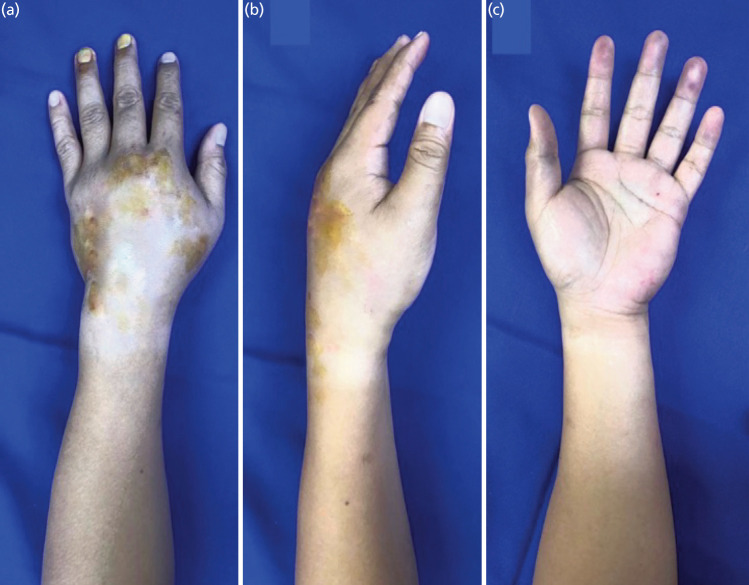
Gross Images of left hand upon arrival at emergency department, (a) dorsal, (b) radial, (c) palmar.

On examination, the patient was ambulatory, conversant, and not in distress. Inspection of the left wrist revealed noticeable swelling, tenderness, and multiple abrasions on the dorsal aspect (Fig. 1). Sensation was intact, and pulses in both the radial and ulnar arteries were full and equal.

**Fig. 2:**
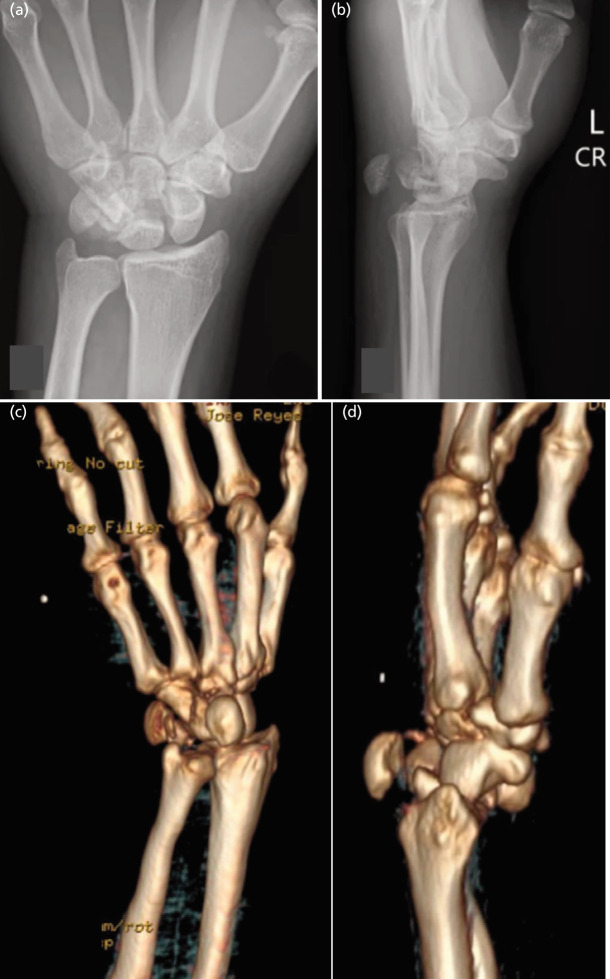
Pre-operative assessment illustrating the closed, complete, transverse, displaced fracture of the body of the left capitate (AO 73A). (a and b) Radiograph of the left hand, and (c and d) 3D reconstruction CT scan of the left hand, highlighting the displacement and misalignment of the capitate bone, confirming the need for open reduction and internal fixation (ORIF) with screw fixation to restore proper anatomical alignment and stability.

Radiographs and CT imaging revealed an isolated, displaced transverse fracture through the body of the capitate (AO 73A) without associated carpal fractures but with notable fragment separation. The 3D reconstruction confirmed normal alignment and no associated fractures or joint incongruities (Fig. 2). Given the severity and displacement, immediate surgical intervention was indicated.

The patient underwent open reduction and internal fixation (ORIF) under regional anaesthesia. A dorsal approach exposed a laterally displaced capitate. A 2.4mm headless compression screw was inserted under fluoroscopic guidance. Intra-operatively, a scapholunate ligament tear was identified and repaired using transosseous suture technique. Fluoroscopy confirmed the alignment of the scaphoid and lunate bones; however, persistent widening between the bones indicated residual instability which warranted a dorsal capsule imbrication. The lax joint capsule was tightened with simple sutures. Once adequate tension was achieved, the capsule was re-approximated.

**Fig. 3:**
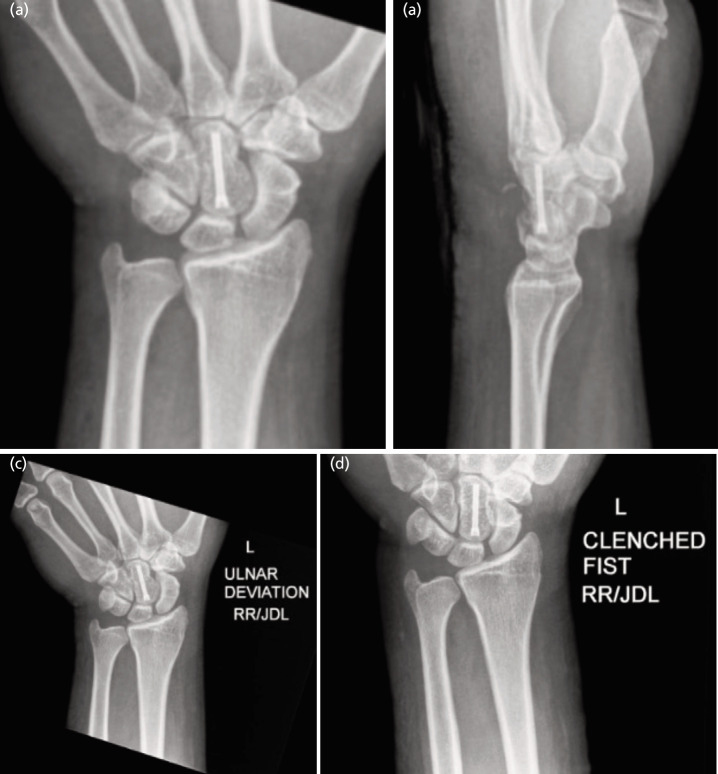
Post-operative radiographs of the hand. (a) PA, (b) lateral, (c) ulnar deviation, (d) clenched fist) confirming the successful reduction and proper alignment of the capitate after open reduction and internal fixation (ORIF) with screw fixation. The PA of the wrist with ulnar deviation and clenched fist demonstrates proper alignment of the scaphoid and lunate, with no evidence of diastasis or abnormal gapping, indicating preserved scapholunate ligament integrity with the scapholunate interval maintained within the normal range of 0.25mm (N .3mm).

The wound was closed in layers and the wrist was immobilised in a splint. Post-operative radiographs confirmed successful reduction and fixation of the capitate, proper repair of the scapholunate ligament, and effective dorsal capsule imbrication, as shown in (Fig. 3).

Rehabilitation began with initial immobilisation to restricted movement and pain control through which slowly progressed to active wrist movements. By week five, dynamic strengthening with moderate resistance and proprioceptive exercises were added. At eight weeks post-surgery, the wrist’s ROM was slightly reduced compared to normal values but still reflected progressive recovery. Grip strength was comparable to the contralateral wrist and the patient reported no pain or functional limitations. By 10 weeks, follow-up radiographs demonstrated fracture union and hardware stability with the patient exhibiting restored wrist ROM and grip strength and returning to full occupational duties.

## Discussion

Capitate fractures rarely occur in isolation since they most often affect their surrounding structures such as perilunate dislocations, scaphoid fractures, or scaphocapitate fracture syndrome^2^. Kadar *et al* provided one of the most comprehensive analyses of capitate fractures, reviewing 53 cases across a 13-year period. In their study, they identified that over 80% of capitate fractures were associated with concomitant wrist or hand injuries, particularly perilunate dislocations, highlighting the importance of assessing for ligament instability once capitate fracture is identified. Furthermore, they reported 11 isolated capitate fractures which were initially managed non-operatively, but 2 required delayed surgical intervention due to non-union^3^.

The management of capitate fractures is dependent on fracture pattern and the presence of associated pathology, ranging from conservative management to surgical intervention. Nondisplaced fractures are generally treated with cast immobilisation, which has shown excellent outcomes due to the capitate's retrograde blood supply from the radial and ulnar arteries^1^. However, displacement or inadequate immobilisation increases the risk of complications such as necrosis, non-union, or arthritis^2^.

A case reported by Lopes and Botton involving a transverse capitate fracture that progressed to non-union due to delayed diagnosis emphasises immediate surgical intervention with ORIF to restore carpal stability and minimise complications such as non-union^4^. In contrast, due to prompt diagnosis, our case was managed within 12 hours post-injury.

Although literature on a complex capitate fracture is limited, existing case reports highlight the benefits of ORIF combined with ligament repair techniques in achieving favourable functional outcomes^3^. ORIF using headless screws or Kirschner wires is a common approach to restore capitate alignment and stability^1^. Given the capitate’s significant role in wrist biomechanics, anatomical reduction is warranted to avoid complications^2^. In our case, a 2.4mm headless screw was used to achieve stable fixation. Thompson *et al* reported similar positive results using the Herbert-Whipple screw demonstrating the efficacy of a rigid internal fixation in restoring alignment and enabling functional recovery^5^.

The scapholunate ligament tear was repaired via transosseous sutures. However, intra-operative imaging showed persistent scapholunate widening despite primary repair prompting capsular reinforcement by dorsal capsular imbrication. Although not as extensive as dorsal capsulodesis, imbrication sufficiently provided additional stability without altering normal carpal kinematics.

The structured post-operative protocol employed in this case emphasised early controlled motion and progressive strengthening in line with best practices following combined osseoligamentous wrist reconstruction. By 10 weeks, the patient regained near-normal wrist motion and grip strength. These findings are consistent with published reports on successful ORIF of capitate fractures when managed appropriately without delay^2,3^.

In conclusion, this case illustrates a rare and complex injury involving a transverse fracture of the capitate, accompanied by disruption of the scapholunate ligament and significant dorsal capsule laxity. The complexity of the case is not commonly encountered in clinical practice. This case report emphasises the importance of a timely, well-planned, and individualised surgical intervention and careful postoperative rehabilitation to ensure optimal functional recovery and long-term wrist stability. This case report contributes to existing literature by bridging knowledge gaps, guiding clinical decision-making, and supporting future investigations into the biomechanics, treatment, and long-term outcomes of isolated capitate fractures.
